# 3-Phenyllactic Acid and Polyphenols Are Substances Enhancing the Antibacterial Effect of Methylglyoxal in Manuka Honey

**DOI:** 10.3390/foods12051098

**Published:** 2023-03-04

**Authors:** Marcus Thierig, Jana Raupbach, Diana Wolf, Thorsten Mascher, Kannan Subramanian, Thomas Henle

**Affiliations:** 1 Chair of Food Chemistry, Technische Universität Dresden, D-01062 Dresden, Germany; 2 Department of Molecular Toxicology, German Institute of Human Nutrition Potsdam-Rehbrücke (DIfE), D-14558 Nuthetal, Germany; 3 Chair of General Microbiology, Technische Universität Dresden, D-01062 Dresden, Germany; 4 Manuka Health New Zealand Limited, 66 Weona Court, Te Awamutu 3800, New Zealand

**Keywords:** manuka honey, *Bacillus subtilis*, methylglyoxal, 3-phenyllactic acid, synergistic effects

## Abstract

Manuka honey is known for its unique antibacterial activity, which is due to methylglyoxal (MGO). After establishing a suitable assay for measuring the bacteriostatic effect in a liquid culture with a time dependent and continuous measurement of the optical density, we were able to show that honey differs in its growth retardingeffect on *Bacillus subtilis* despite the same content of MGO, indicating the presence of potentially synergistic compounds. In model studies using artificial honey with varying amounts of MGO and 3-phenyllactic acid (3-PLA), it was shown that 3-PLA in concentrations above 500 mg/kg enhances the bacteriostatic effect of the model honeys containing 250 mg/kg MGO or more. It has been shown that the effect correlates with the contents of 3-PLA and polyphenols in commercial manuka honey samples. Additionally, yet unknown substances further enhance the antibacterial effect of MGO in manuka honey. The results contribute to the understanding of the antibacterial effect of MGO in honey.

## 1. Introduction

Honey is a food item known for its antibacterial activity, which is due to physical factors such as high osmolarity and low pH value as well defined antibacterial compounds such as hydrogen peroxide, produced by glucose oxidase, and antibacterial peptides from the honeybee such as “bee-defensin” [1,2,3]. Compared with “conventional” honeys, such as linden or rapeseed honey, the pronounced antibacterial activity of manuka honey (*Leptospermum scoparium*) is due to high concentrations of methylglyoxal (MGO), which is formed by dehydration of dihydroxyacetone in the nectar during maturation [4,5]. While MGO is present in conventional honeys in amounts up to 24.1 mg/kg [6], the MGO levels in manuka honey reach up to 1541 mg/kg, depending on the location of the bee hive [7,8,9]. On the other hand, manuka honey is classified as a “low-peroxide” honey due to its low glucose oxidase activity [10].

Besides the use as a food item, honey and, especially manuka honey, is used as a non-adherent dressing in medical wound management, in which the mentioned properties help to stimulate tissue regeneration and to keep the wound sterile [11,12]. A standard method for the determination of the antibacterial activity of (manuka) honey is the agar diffusion assay. Thereby, honey solution is placed into a cavity of an agar plate and inoculated with a culture of *Staphylococcus aureus*. After 24 h of incubation, the antibacterial effect of the honey solution can be estimated based on the inhibition zones around the samples and by comparing the effect caused by the honey samples with standard solutions of an antibacterial agent, e.g., phenol [13,14]. This technique has the drawback that the agar plates cannot be evaluated precisely and that no statements about bacteriostatic or bactericidal effects are possible. For the classification of the antibacterial activity of manuka honey, Swift et al. (2014) established a growth assay in liquid cultures, in which it has been shown that MGO has a bacteriostatic effect on *S. aureus* [15]. Besides that, a quantitative evaluation of the antibacterial effect of MGO in honey on specific bacteria can be found rarely in the literature, as well as with respect to the variety of bioactive substances in honey besides MGO, such as hydrogen peroxide [1].

Additionally, it has already been shown that MGO and manuka honey have a synergistic effect on the antibacterial and antiviral activity of antimicrobial substances. For example, the addition of linezolid, an oxazolidinone-based antibiotic inhibiting the protein biosynthesis of Gram-positive bacteria, reduced the MIC of MGO against *S. aureus* in a checkerboard broth microdilution assay by a factor of four in concentrations below its minimal inhibitory concentration (MIC) [16]. Similar effects were observable for *S. aureus* biofilms when adding Rifampicin to manuka honey or on influenza viruses (H1N1) in Madin–Darby canine kidney cells when adding the antiviral agents Zanamivir or Oseltamivir to manuka honey [17,18]. On the other hand, little can be found in the literature on whether the antibacterial effect of MGO itself can be enhanced by naturally occurring compounds present in manuka honey. It has been shown that the growth delay of *S. aureus* and *Pseudomonas aeruginosa* were higher when treated with manuka honey in concentrations above 250 mg/kg and α-cyclodextrin, compared with the honey solutions without α-cyclodextrin [15]. Furthermore, the only substances native to (manuka) honey discussed to have an antibacterial effect themselves are syringic acid and 3,4,5-trimethoxybenzoic acid [19]. Manuka and non-manuka honeys also contain polyphenolic compounds in concentrations up to 2967 mg gallic acid equivalents (GAE)/kg, which provide an antioxidant activity in the honey [20,21]. Synthetic derivatives of gallic acid show an antibacterial activity against *E. coli*, *S. aureus* and *Bacillus subtilis* [22]. So far, it is unknown if syringic acid, 3,4,5-trimethoxybenzoic acid or naturally occurring polyphenolic compounds enhance the effect of MGO in manuka honey. Since manuka honey is also rich in other organic acids with structural similarities, such as 3-phenyllactic acid (3-PLA), 4-hydroxyphenyllactic acid and 2-methoxybenzoic acid, we hypothesized that these substances can influence the antibacterial effect of manuka honey. As 3-PLA is a marker substance for manuka honey, manuka honey has to contain at least 500 mg/kg 3-PLA per definition [23]. Due to the high 3-PLA contents up to 1400 mg/kg in manuka honey, which are also in the same order of magnitude as MGO, and its relevance as a marker substance for manuka honey, it will be considered in this study [24]. Therefore, the aim of this study was to evaluate possible synergistic effects of MGO, 3-PLA and polyphenols on the antibacterial activity of manuka honey.

## 2. Materials and Methods

### 2.1. Honey Samples

In this study, four commercially available manuka honeys, all labeled for the purpose of wound healing treatments; two manuka honey labeled with MGO250+ (containing 270 mg/kg MGO) and MGO400+ (containing 444 mg/kg MGO) and a cornflower honey, as references; and a manuka honey labeled with MGO30+ (containing 72 mg/kg MGO) as a basis for spike experiments were included. After purchase, all samples were stored at 4 °C until analysis. As a blank matrix and for dilution of the honey samples, artificial honey prepared following Deng et al. (2018) was used in the assay to obtain constant osmotic pressure [25]. In brief, 44 g of fructose, 37 g of glucose and 2 g sucrose were dissolved in 17 g water. The suspension was slightly heated to 45 °C and stirred until full dissolved.

### 2.2. Chemicals

Methanol (HPLC grade) and acetonitrile (LC-MS grade) were purchased from VWR (Darmstadt, Germany). Methylglyoxal, ortho-phenylenediamine, 2-methylchinoxaline, gallic acid, 3-phenyllactic acid, forchlorfenuron, 1,3-dihydroxyacetone-dimer and Folin–Ciocalteu-reagent were obtained from Sigma Aldrich/Merck (Steinheim, Germany). Formic acid, acetic acid, fructose, glucose, sucrose and LB-broth were obtained from Carl Roth (Karlsruhe, Germany). Sodium carbonate, sodium dihydrogenphosphate and disodium hydrogenphosphate were purchased from Grüssing (Filsum, Germany). 3-Deoxyglucosone (3-DG) was synthesized according to Henle and Bachmann (1996) [26]. Double-distilled water (Bi 18E double distillation system, QCS, Maintal, Germany) was used for HPLC solvents.

### 2.3. Quantification of MGO in Manuka Honeys and in Model Solutions

The quantification of MGO was performed according to Atrott et al. (2012) with slight modifications [5]. Briefly, 150 µL ortho-phenylenediamine (OPD) solution (1% in phosphate-buffered saline (PBS)) was added to 650 µL of a 2.5% solution on honey in 0.5 M PBS with pH 6.5 or to a mixture of 150 µL model solution and 500 µL PBS, respectively, for an overnight incubation at room temperature in the absence of light. After membrane filtration (0.45 µm), the samples were analyzed via HPLC-UV. The analyses were performed using a system containing a pump, including an online-degasser and a mixing chamber P 6.1 L, an autosampler AS 6.1 L, a column thermostat CT 2.1 and a diode array detector DAD 2.1 L, all from Knauer (Berlin, Germany). The separation of the quinoxalines was achieved on a column filled with Eurospher C-18 material (250 × 4.6 mm, 5 µm particle size, with integrated precolumn, Knauer, Berlin, Germany) as the stationary phase and a mobile phase containing 0.075% acetic acid as solvent A and a mixture of 20% solvent A and 80% methanol as solvent B. The gradient started with 40% solvent B for 1 min, was elevated to 100% B within 20 min, was changed back to 40% B in 4 min and was held there for an additional 5 min. The separation was performed with a flow rate of 0.9 mL/min; at a temperature of 30 °C, 20 µL of the sample was injected. The detection of the peaks was conducted by measuring the UV-absorbance at 312 nm. Quantification was achieved with an external calibration with commercial MGO solution, of which the MGO content was determined after derivatization with OPD by comparison with 2-methylquinoxaline standard.

### 2.4. Extraction of Honey Proteins

To extract the high molecular fraction of the honeys containing the honey protein, honey samples were prepared according to the protocol published by Hellwig et al. (2017), with slight modifications [27]. Approx. 5 g of honey was dissolved in 10 mL of water and transferred into a dialysis tube (MWCO 14 kDa, Sigma, Steinheim, Germany), followed by dialysis against water. During the 2 days of dialysis, the water was changed twice a day. Afterwards, the retentates were freeze-dried and stored at −18 °C until sampling. About 20 mg of honey protein was obtained of each sample.

### 2.5. Quantification of 3-Phenyllactic Acid in Manuka Honeys

To determine the content of 3-phenyllactic acid, the honey samples were prepared according to the protocol published by the NZ Ministry for Primary Industries (2017) with slight modifications [28]. Briefly, 1.5 g honey was dissolved in 10 mL of a solution consisting of acetonitrile, formic acid and water (10:1:90 *v*/*v*). The samples were shaken for 20 min with an overhead shaker until complete dissolution. After centrifugation (3000 g, 10 min) and membrane filtration (0.45 µm), 50 µL solution was mixed with 940 µL extraction solution and 10 µL internal standard solution (forchlorfenuron, 10 mg/L in 1% formic acid in acetonitrile). This solution was injected in the HPLC-MS/MS system, consisting of a binary pump G1312A, an online-degasser G1379B, an autosampler G1329A, a column thermostat G1316A and a mass spectrometer with electrospray ion source G6410A, all from Agilent Technologies (Santa Clara, CA, USA). Separation was achieved with a Kinetex 100 C18 column (2.1 × 50 mm, 1.7 µm, with precolumn, Phenomenex, Torrance, CA, USA) as a stationary phase and a mobile phase containing 0.1% formic acid in water as solvent A and 0.1% formic acid in acetonitrile as solvent B. The gradient started with 5% solvent B for 0.75 min, was elevated to 15% B within 1.25 min and 70% B within 2 min before a final increase to 98% B within 2 min. The concentration was held for 2 min, changed back to 5% B within 1 min and finally held for 7 min. The separation was performed with a flow rate of 0.2 mL/min at a temperature of 40 °C. In total, 5 µL of the sample was injected. For quantification, MRM transitions (see Table 1) were recorded and an external calibration with standard solutions of 3-phenyllactic acid (concentrations between 0.025 and 1 mg/L) was used. General working conditions of the mass spectrometer were 350 °C gas temperature, 11 L/min gas flow, 35 psi nebulizer pressure and 4000 V capillary voltage.

### 2.6. Estimation of the Polyphenol Content in Honey via Folin–Ciocalteu Method

The determination of total polyphenols was achieved using the method of Singleton et al. (1999), with slight modifications [29]. In total, 20 µL of sample solution (15% *w*/*v* in water) or calibration solution (gallic acid, dissolved in water, concentrations ranging between 5 and 200 mg/L) and 100 µL of 0.2 N Folin reagent solution (Sigma Aldrich, Steinheim, Germany) were pipetted into a cavity of a 96-well plate. After 5 min, 80 µL of a 75 g/L sodium carbonate solution was added. After a reaction time of 120 min, the absorbance of the resulting blue dye was measured at 760 nm against a blank value containing water instead of sample or calibration solution.

### 2.7. Determination of the Bacteriostatic Effect of Manuka Honey Solutions against Bacillus subtilis

The determination of the bacteriostatic effect of manuka honey solutions against *B. subtilis* W168 was performed according to Jenkins and Cooper (2012), with modifications [30]. Therefore, an overnight culture was prepared by inoculating approx. 10 mL liquid LB-broth with a colony forming unit of the bacterial strain. The next day, the OD_600_ was measured of a 1:10 (*v*/*v*) solution of the culture against LB-broth with a spectrophotometer UV-3100PC (VWR, Darmstadt, Germany). To evaluate the antibacterial effect, 30% solutions of the honey samples or of artificial honey, respectively, were prepared with liquid LB-broth and sterile filtered (0.2 µm). To realize different amounts of methylglyoxal in the samples, while maintaining the sugar concentration at the same level, honey solutions were diluted with artificial honey solution. This also ensured a constant osmotic pressure in each diluted sample. For the assay, 105 µL of the diluted or undiluted honey sample solution was pipetted into a cavity of a 96-well plate. In total, 105 µL of the solution of artificial honey was used as blank. An aliquot of the 1:10 diluted overnight culture was placed into each cavity, such that the resulting OD_600_ was calculated to be 0.05. Finally, LB-broth was added to make a volume of 210 µL, such that the assay solution contained 15% (*w*/*v*) in total. To prove sterile conditions, 210 µL of liquid LB-broth was tested as well as blank. To obtain growth curves, the samples were incubated at 37 °C for 24 h while being shaken continuously. The OD_600_ was measured every 5 min using a Biotek EPOCH 2 microplate reader (Agilent, Santa Clara, CA, USA). To calculate the bacteriostatic effect, the end of the bacterial lag phase was defined as the OD_600_ value 5-fold higher than the initial OD_600_ value. Assuming a proportionality between the OD_600_ and the cell count, this factor of 5 corresponds to two-generation growth cycles of the bacteria. To calculate a growth arrest or delay, the time of the lag phase measured for bacterial growth in the honey sample was divided by the lag time obtained for the bacterial growth in artificial honey on the respective 96-well plate as control. The calculated growth delay gives a statement about the bacteriostatic effect, i.e., the increase in the duration of the lag-phase due to the presence of antibacterial compounds. If the bacteria did not grow during the whole measurement, the effect was defined to be bactericidal.

## 3. Results and Discussion

### 3.1. Measurement of the Antibacterial Activity of Honeys

**Figure 1:**
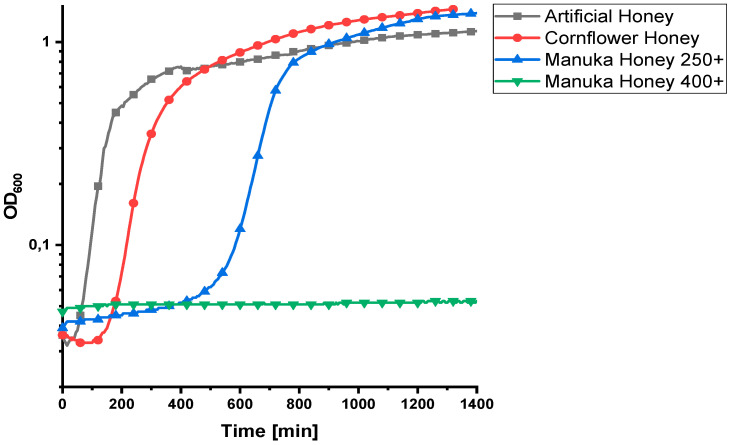
Growth curves of *B. subtilis* treated with artificial honey, cornflower honey, manuka honey MGO250+ and manuka honey MGO400+.

To evaluate the antibacterial effect of MGO, *Bacillus subtilis* was chosen as a bacterial model strain. Due to the absence of glutathione in cells of *B. subtilis*, and the production of bacillithiol instead, it has a lower capacity to detoxify MGO [31]. Therefore, enzymatic intracellular MGO degradation can be neglected. Additionally, the antibacterial activity of hydrogen peroxide formed by glucose oxidase in honeys can be ruled as an antibacterial factor since *B. subtilis* is able to degrade hydrogen peroxide due to its catalase activity. These assumptions were tested by measuring growth curves of *B. subtilis* in presence of 15% solutions of artificial honey, cornflower honey and manuka honeys labeled MGO250+ and MGO400+, respectively. Cornflower honey, which is well known for a high glucose oxidase activity, showed a slight inhibiting effect with a growth delay of 2.3 when compared with artificial honey (see Figure 1). Furthermore, the addition of hydrogen peroxide to artificial honey in honey-relevant concentrations did not lead to a delayed growth of *B. subtilis* significantly (see Figure S1). In contrast, manuka honey MGO250+ showed a growth delay of 5.3. This confirms that hydrogen peroxide only plays a minor role in the antibacterial effect of honey on *B. subtilis*, and MGO in manuka honey can be assumed as the main inhibiting compound. The inhibiting effect of manuka honey MGO250+ on the growth of *B. subtilis* is clearly observable (Figure 1).

The strength of the antibacterial effect is dependent on the MGO content of the investigated honeys. Higher MGO contents lead to longer a lag phase, after which the bacteria start to grow. The bacteriostatic, but not bactericidal, effect is likely due to chemical or microbial degradation of MGO during the measurement. MGO is able to react within the Maillard reaction with lysine and arginine side chains of the proteins in liquid medium [32], which leads to a reduction of the MGO content. Besides its weak glyoxalase system, *B. subtilis* may degrade MGO via other pathways, e.g., with aldo-keto reductase to acetol [33]. If the MGO level drops below a certain concentration, the bacteria are able to start growing. On the other hand, in the presence of MGO400+ honey bacteria did not grow during the measurement. For the purpose of this study, this is considered to be a bactericidal effect, even though it cannot be ruled out that the strain would have grown after a longer incubation period.

Therefore, this model allows quantification of the antibacterial activity of different honeys. In particular, comparative statements between (manuka) honeys are possible, especially when compared with a reference honey. Principally, this assay is also transferable to other bacterial strains. However, for the evaluation of the bacteriostatic effect, the activity of glyoxalase, catalase and other enzymes responsible for the degradation of antibacterial compounds have to be considered.

### 3.2. Antibacterial Activity of Commercial Manuka Honey Samples

**Figure 2:**
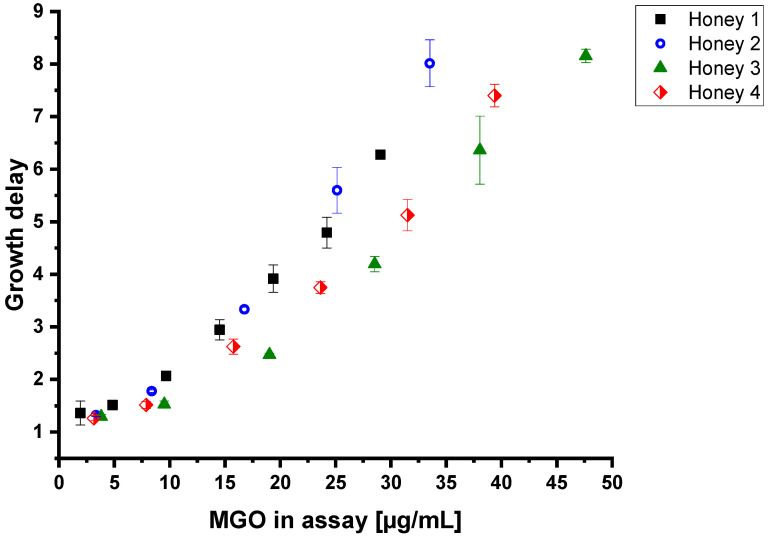
Growth delays of *B. subtilis* treated with four different manuka honeys in dependence of the MGO content in the assay after dilution to 1%, 2.5%, 5%, 7.5%, 10%, 12.5% and 15% (*w*/*v*) with 30% artificial honey solution and LB-medium. Measurements were carried out in triplicates. Error bars show standard deviation.

To evaluate the antibacterial activity of commercial manuka honey samples, the assay was applied to four commercial manuka honeys which were labeled for wound healing purposes. Besides using the 30% honey solutions for measurements (resulting in a 15% solution in the assay), all honeys were also diluted to 2%, 5%, 10%, 15% and 20% with a 30% solution of artificial honey in liquid medium prior to the addition of the liquid culture and LB-medium. The dilution was obtained with artificial honey to achieve varying MGO levels but to keep constant osmotic pressure in the assays. Thereby, every sample contains the same amount of sugars, which is necessary to compare the antibacterial effect of diluted honey samples [3]. It can be seen that higher MGO contents in the assay lead to higher growth delays (Figure 2, Table S1). In addition, it is noticeable that similar contents of MGO in the honeys did not necessarily lead to the same growth delays and, conversely, no conclusion can be drawn from the growth delay to the MGO content in the assay. Comparing honey one with honey three, for instance, an adjustment to 30 µg MGO per mL (corresponding to a 30% solution of a honey containing 100 mg/kg MGO) resulted in GD values of four and six, respectively. In reverse, to obtain a growth delay of five, a MGO concentration of 24 µg/mL of honey one or 31 µg/mL of honey four is needed.

As mentioned above, hydrogen peroxide as a second antibacterial agent in honeys can be excluded due to active catalase in the bacterial cell. Additionally, different pH values of the honeys can also be excluded, since measurements of the antibacterial activity of artificial honey spiked with MGO (pH~6.5) did not differ when the pH value of artificial honey was adjusted to five with gluconic acid. Therefore, neither the glucose oxidase activity nor the pH values of the honeys explain the differences in growth delay. The effect of MGO on bacterial growth has to be enhanced or reduced by other honey ingredients.

### 3.3. Determination of Synergistic Effects in Manuka Honey

The next aim of the study was to analyze which compounds, besides MGO, might be relevant for the antibacterial activity. Therefore, artificial honey spiked with MGO was used as a model system, to which potential synergistic substances were added in honey at relevant concentrations. The following substances were chosen as potential synergists: dihydroxyacetone (DHA) as the precursor substance of MGO in manuka honey; isolated manuka honey protein; gallic acid as a representative for phenolic compounds in honey; 3-phenyllactic acid (3-PLA) as a marker substance of manuka honey, occurring in similar concentrations as MGO and considered to have antibacterial effects on Gram-positive bacteria [34]; and 3-desoxyglucosone (3-DG) as another abundant dicarbonyl compound in honey.

Except for 3-PLA, none of the compounds tested were found to enhance the antibacterial activity of MGO (see Figures S2 and S3 in the supplementary material). Honey protein especially did not show any particular effect. For further studies, structural changes and a loss of a possible antibacterial effect during the protein extraction should be considered.

**Figure 3:**
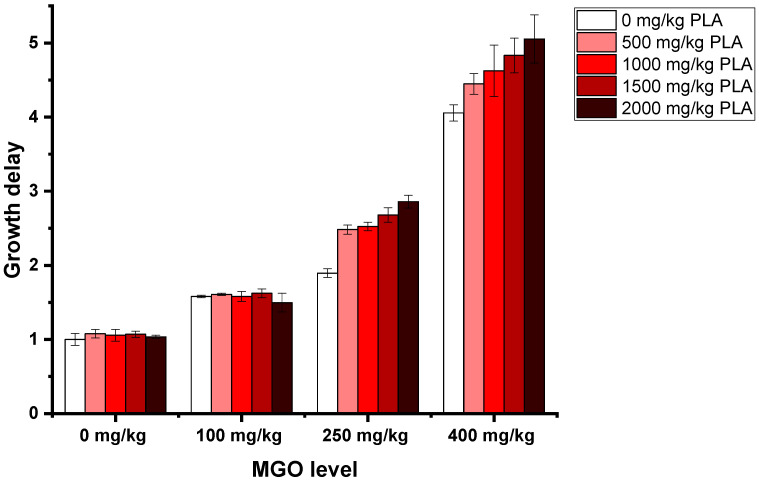
Growth delays of *B. subtilis* treated with 15% (*w*/*v*, in LB medium) solutions of artificial honey; artificial honeys were spiked with MGO and 3-PLA. Measurements were carried out in quadruplicates. Error bars show standard deviation.

Whereas 3-PLA in honey-relevant concentrations ranging up to 2000 mg/kg alone or added to artificial honey containing 100 mg/kg MGO did not lead to a delay in the growth curves, adding 3-PLA to artificial honeys with 250 mg MGO/kg or higher clearly increased the antibacterial activity caused solely by MGO. As an example, the addition of 2000 mg/kg 3-PLA to artificial honey with 400 mg/kg MGO increased the growth delay from 4.06 to 5.05. (Figure 3, Table S2).

**Figure 4:**
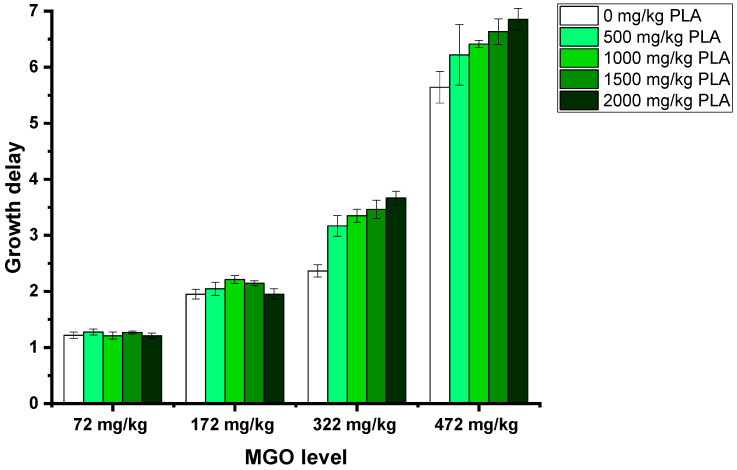
Growth delays of *B. subtilis* treated with 15% (*w*/*v*, in LB medium) solutions of manuka honey MH30+; honeys were spiked with MGO and 3-PLA. Measurements were carried out in quadruplicates. Error bars show standard deviation.

To simulate real honey samples, the experiment was repeated with a manuka honey naturally containing 72 mg/kg MGO. This manuka honey was spiked with different amounts of MGO up to an additional 400 mg/kg and 3-PLA up to 2000 mg/kg. Concerning the antibacterial activity, the same effect was observed as in the artificial honeys: the addition of 3-PLA resulted in a dose-dependent increased growth delay when MGO concentrations of 322 mg/kg or higher were present, e.g., the addition of 2000 mg/kg 3-PLA to the honey containing 472 mg/kg MGO increased the growth delay from 5.64 to 6.85 (Figure 4, Tables S2 and S4). In the presence of lower MGO concentrations, 3-PLA did not show a growth delay enhancing effect.

There are two explanatory approaches for the synergistic effect of 3-PLA with MGO. Firstly, MGO is stabilized by 3-PLA in the medium. MGO is a reactive substance which can react with proteins in the liquid medium. Therefore, the MGO concentration in the assay decreases over time. To test this, MGO was diluted to a concentration of 120 mg/L (corresponding to a 30% solution of a honey containing 400 mg/kg MGO) with LB medium and in LB medium containing 600 mg/L 3-PLA (corresponding to a 30% solution of a honey containing 2000 mg/kg 3-PLA). The samples were incubated without the presence of *B. subtilis*. The MGO content was analyzed at 0 h, 1 h, 3 h, 5 h, 8 h and 24 h during the 24 h incubation at 37 °C.

**Figure 5:**
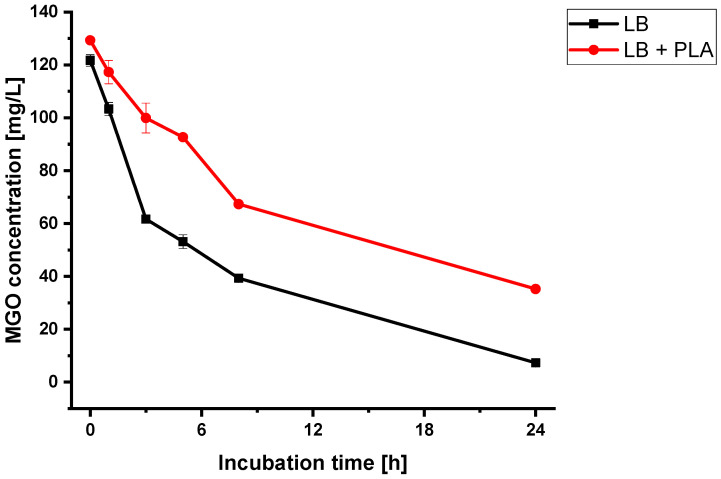
MGO concentrations during the incubation (24 h at 37 °C) of 120 mg/L MGO solution in LB medium (LB) and in LB medium containing 600 mg/L 3-PLA (LB + PLA) (without *B. subtilis*). Measurements were carried out in triplicates. Error bars show standard deviation.

While the MGO level in the sample without 3-PLA dropped from 120 mg/L to 7 mg/L within 24 h, the MGO content in the sample with 3-PLA decreased to 35 mg/L (Figure 5, Table S3). Therefore, a higher apparent MGO concentration in the assay leads to longer lag-times of the bacteria. The mechanism of how MGO is stabilized by 3-PLA is still unknown.

Besides an extracellular effect, intracellular effects might be relevant as well for the synergistic effect of 3-PLA. It has been shown in the literature that 3-PLA in high concentrations (>10 mg/mL) damages or alters the cell wall of Gram-positive bacteria, e.g., *Listeria monocytogenes,* due to the loss of cell wall rigidity [34]. With regard to manuka honey, 3-PLA might also interact with bacterial cell walls in honey-relevant concentrations (~100 µg/mL) without leading to cell death, but to “softening” the cell wall. This could lead to a higher susceptibility of the cell towards MGO by increasing intracellular MGO concentrations.

**Figure 6:**
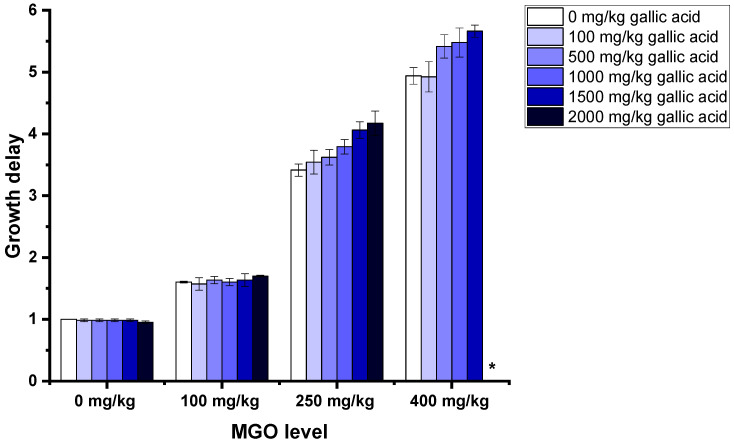
Growth delays of *B. subtilis* treated with 15% (*w*/*v*, in LB medium) solutions of artificial honey; artificial honeys were spiked with MGO and gallic acid. The star indicates the bactericidal effect of this mixture. Measurements were carried out in duplicates. Error bars show standard deviation.

Besides 3-PLA, gallic acid showed similar properties enhancing the antibacterial effect of MGO. When added to artificial honeys containing no MGO, gallic acid did not have a growth delaying effect. On the other hand, when added to artificial honey containing 250 mg MGO/kg or more, higher levels of gallic acid lead to a higher growth delay at the same MGO concentration (Figure 6). Additionally, in the artificial honey containing 400 mg/kg MGO, the increase in gallic acid from 1500 mg/kg to 2000 mg/kg resulted in a bactericidal effect. In this assay, gallic acid was used as a representative for the polyphenolic compounds in (manuka) honey. Despite the fact that honeys contain rather small amounts of up to 66 mg/kg of gallic acid [35], the content of polyphenols expressed as gallic acid equivalents (GAE) is within the range of our model honeys, as manuka honeys containing 2170 mg GAE/kg have been described in the literature [20].

In order to check whether these results are an explanation for the differences in the antibacterial properties between honeys with similar MGO contents (Figure 2), the contents of 3-PLA and polyphenols, expressed as gallic acid equivalents (GAE), were measured (Table 2).

It is noticeable that the two honeys with the highest 3-PLA and GAE content are also the honeys with the highest growth delay against *B. subtilis*. As an example, to obtain a growth delay of about five with “honey 1” (containing 734 mg/kg 3-PLA and 636 mg/kg GAE), a MGO concentration of 24 µg/mL is needed, while “honey 4” (334 mg/kg PLA and 386 mg/kg GAE) obtains a growth delay of five at 31 µg/mL, which might be due to different amounts of 3-PLA and GAE in the assay. Nevertheless, the exact quantitative contribution to the antibacterial effect of manuka honey, especially in models containing more than one synergist, and the specific mechanism of action of the synergistic effect have to be investigated in further studies.

**Figure 7:**
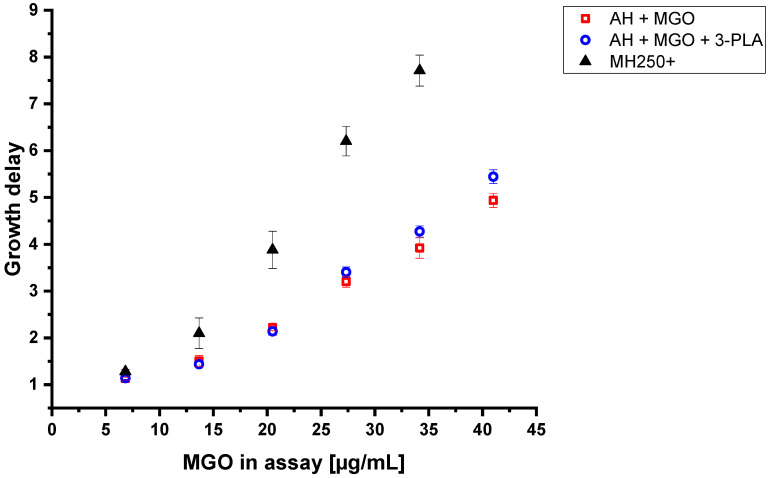
Growth delays of *B. subtilis* treated with artificial honey spiked with 259 mg/kg MGO (AH + MGO), artificial honey spiked with 259 mg/kg MGO and 467 mg/kg PLA (AH + MGO + 3-PLA) and commercial MH250+ manuka honey with 259 mg/kg MGO and 467 mg/kg PLA (MH250+) in dependence on the MGO content in the assay after dilution to 1%, 2.5%, 5%, 7.5%, 10%, 12.5% and 15% (*w*/*v*) with 30% artificial honey solution and LB-medium. Measurements were carried out in triplicates. Error bars show standard deviation.

To verify the synergistic effect of 3-PLA, the antibacterial activities of a manuka honey containing 259 mg/kg MGO and 467 mg/kg 3-PLA, an artificial honey spiked to the same MGO concentration and another spiked artificial honey with the same MGO and 3-PLA concentrations were compared. It was confirmed that 3-PLA enhances the effect of MGO above a concentration of 34.1 µg/mL, corresponding to a 30% solution of a honey containing 113 mg/kg MGO. (Figure 7, Table S5)

Restrictively, this enhancing effect is not enough to reach the antibacterial level of the commercial manuka honey sample. Whereas a MGO concentration of 27.3 µg/mL obtained by MGO-spiked artificial honey leads to a growth delay of 3.2 without 3-PLA and 3.4 in the presence of 3-PLA, the same MGO level obtained by manuka honey leads to a growth delay of 6.2 (Figure 7). Therefore, it can be concluded that polyphenolic compounds, as well as additional compounds, presumably other organic acids such as 4-hydroxyphenyllactic acid, 2-methoxybenzoic acid, syringic acid or 3,4,5-trimethoxybenzoic acid, or yet unknown substances in manuka honey, enhance the effect of MGO.

## 4. Conclusions

For the first time, we demonstrated that 3-phenyllactic acid, a marker compound of manuka honey occurring in concentrations up to 1400 mg/kg, enhances the antibacterial activity of artificial honeys containing 250 mg/kg MGO or more against the model bacterium *B. subtilis*, even if 3-phenyllactic acid alone does not show any bacteriostatic effect in honey-relevant concentrations. This is due to higher apparent MGO concentrations in the assay due to MGO stabilization by 3-PLA. Additionally, first results indicate that polyphenols, tested with gallic acid as a substitute with similar chemical properties, also enhance the antibacterial effect of MGO. Therefore, 3-PLA and polyphenols as synergists of MGO may also be considered for the qualitative evaluation of manuka honey besides their chemical benefits. Nevertheless, the differences in the antibacterial activities between artificial honey spiked with MGO and commercial manuka honey with the same amount of MGO cannot be explained uniquely by 3-PLA and GAE. Other substances in (manuka) honey appear to enhance the effect of MGO as well.

The results of this study contribute to the understanding of the antibacterial mechanism of MGO itself and in manuka honey. The results further support the application of manuka honey beyond its use as food item, e.g., to the use of manuka honey for functional and medical applications such as wound healing. However, the assay presented in this study should be transferred to other bacteria, especially those without a proper glyoxalase system, such as *S. aureus* [31], as well as to other Gram-positive and Gram-negative bacteria and other microbiota to obtain more general data about the mechanism of action of the antimicrobial activity of methylglyoxal and manuka honey.

## Figures and Tables

**Table 1:** Transitions recorded during MRM Measurement of 3-phenyllactic acid (3-PLA) and the internal standard forchlorfenuron (FCF) in honey samples.

Compound	Ionization	Precursor Ion [*m/z*]	Product Ion [*m/z*]	Fragmentor Voltage [V]	Collision Energy [eV]	Dwell Time [ms]	Q/q ^a^
3-PLA time segment 5–8.7 min	negative	165	147	50	5	100	Q
		165	119		15		q
		165	103		15		q
FCF time segment 8.7–16 min	positive	248	129	100	10	100	Q
		248	93		40		q

^a^ Q: transition used for quantification, q: transition used for identification.

**Table 2:** Contents of methylglyoxal, 3-phenyllactic acid and gallic acid equivalents in the analyzed honey samples. Measurement uncertainty is given as standard deviation (*n* = 3).

	Methylglyoxal [mg/kg]	3-Phenyllactic Acid [mg/kg]	Gallic Acid Equivalents [mg/kg]
Honey 1	194 ± 2	734 ± 6	636 ± 43
Honey 2	335 ± 3	674 ± 16	795 ± 36
Honey 3	381 ± 1	352 ± 13	530 ± 45
Honey 4	315 ± 1	334 ± 5	386 ± 17

## Data Availability

Data are contained within the article and the supplementary materials.

## Supplementary Materials

The following supporting information can be downloaded at: https://www.mdpi.com/article/10.3390/foods12051098/s1, Table S1: Raw data of Figure 2: MGO contents and growth delays of *B. subtilis* in the assay for the determination of the antibacterial activity of different manuka honeys; Table S2: Raw data for Figure 3 and Figure 4: Growth delays of *B. subtilis* treated with artificial honey or manuka honey MH30+ spiked with MGO and 3-PLA; Table S3: Raw data of Figure 5: MGO concentrations during the incubation (24 h at 37 °C) of LB medium containing MGO and LB medium containing MGO and 3-PLA; Table S4: Raw data of Figure 4: Growth delays of *B. subtilis* treated with artificial honeys containing MGO and gallic acid; Table S5: Growth delays of *B. subtilis* treated with artificial honey spiked with MGO, artificial honey spiked with MGO and 3-PLA and manuka honey MH250+; Figure S1: Growth curves of *B. subtilis* treated with LB medium spiked with hydrogen peroxide; Figure S2: Growth delays of *B. subtilis* treated with artificial honeys containing MGO and isolated honey proteins; Figure S3: Growth delays of *B. subtilis* treated with artificial honeys containing MGO, 3-deoxyglucosone or dihydroxyacetone.
