# Supplementary material for: 3-Phenyllactic Acid and Polyphenols Are Substances Enhancing the Antibacterial Effect of Methylglyoxal in Manuka Honey
Source: Foods. 2023 Mar 4;12(5):1098. doi: 10.3390/foods12051098 (PMC10000891; doi:10.3390/foods12051098)
Supplement: Supplementary file 1 [file foods-12-01098-s001.zip › foods-2206555-supplementary.pdf]

3-Phenyllactic Acid and Polyphenols are Substances Enhanc-ing the Antibacterial Effect of Methylglyoxal in Manuka Honey

Marcus Thierig [1], Jana Raupbach [1,2], Diana Wolf [3], Thorsten Mascher [3], Kannan Subramanian [4], Thomas Henle[1,*]

[1] Chair of Food Chemistry, Technische Universität Dresden, D-01062 Dresden, Germany
[2] Department of Molecular Toxicology, German Institute of Human Nutrition Potsdam-Rehbrücke (DIfE), D-14558 Nuthetal, Germany
[3] Chair of General Microbiology, Technische Universität Dresden, D-01062 Dresden, Germany
[4] Manuka Health New Zealand Limited, 66 Weona Court, Te Awamutu, New Zealand

* Correspondence: thomas.henle@tu dresden.de

Supplementary materials

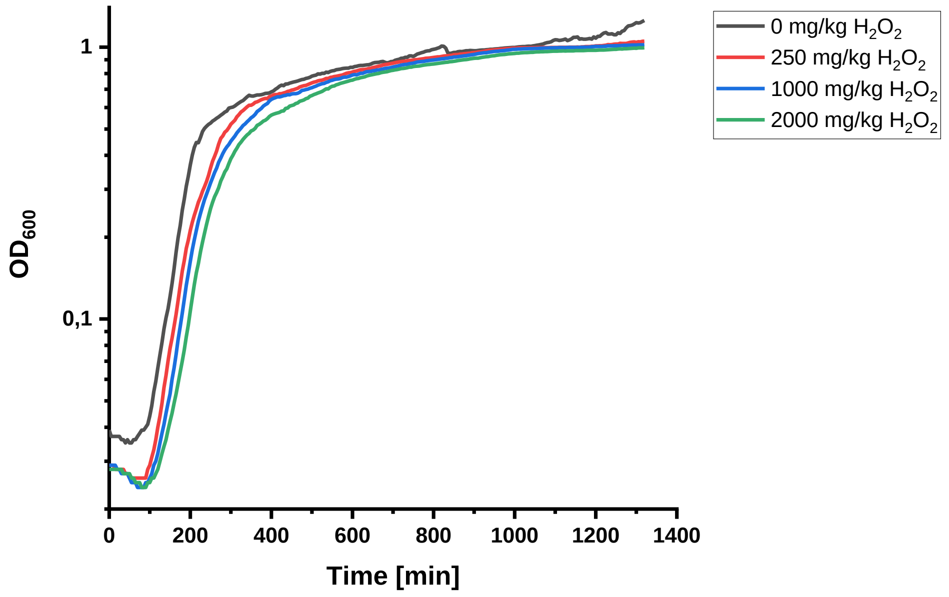

Figure S1. Growth curves of *B. subtilis* treated with LB-medium spiked with hydrogen peroxide

Table S1. Raw data of figure 2: MGO contents and growth delays (GD) in the assays for the determination of the antibacterial activity of different Manuka honeys against *B. subtilis*. Measurements were carried out in triplicates. Measurement uncertainty shows standard deviation.

Honey dilution	Honey 1		Honey 2		Honey 3		Honey 4	
	MGO [µg/mL]	GD	MGO [µg/mL]	GD	MGO [µg/mL]	GD	MGO [µg/mL]	GD
1 %	1.94	1.36 ± 0.23	3.35	1.32 ± 0.02	3.81	1.29 ± 0.04	3.15	1.26 ± 0.05
2.5 %	4.85	1.51 ± 0.09	8.38	1.78 ± 0.02	9.53	1.53 ± 0.06	7.88	1.51 ± 0.06
5 %	9.70	2.06 ± 0.09	16.75	3.33 ± 0.04	19.05	2.47 ± 0.02	15.75	2.63 ± 0.14
7.5 %	14.55	2.94 ± 0.19	25.13	5.60 ± 0.43	28.58	4.19 ± 0.15	23.63	3.75 ± 0.11
10 %	19.40	3.92 ± 0.26	33.50	8.01 ± 0.45	38.10	6.36 ± 0.65	31.50	5.13 ± 0.30
12.5 %	24.25	4.79 ± 0.29	41.88	no growth	47.63	8.15 ± 0.13	39.38	7.40 ± 0.21
15 %	29.30	6.28 ± 0.02	50.25	no growth	57.15	no growth	47.25	no growth

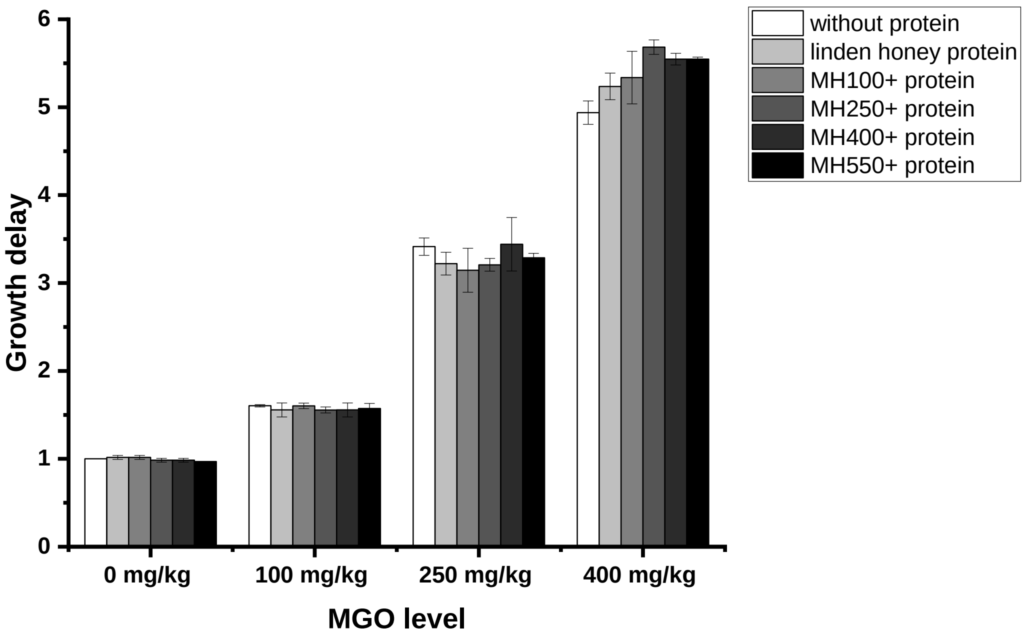

Figure S2. Growth delays of *B. subtilis* treated with 15 % (w/v, in LB medium) solutions of artificial honey, artificial honeys were spiked with MGO and no further protein or 0.1 % (w/v) protein extracted from linden honey and manuka honeys MH100+, MH250+, MH400+, and MH550+. Measurements were carried out in duplicates. Error bars show standard deviation.

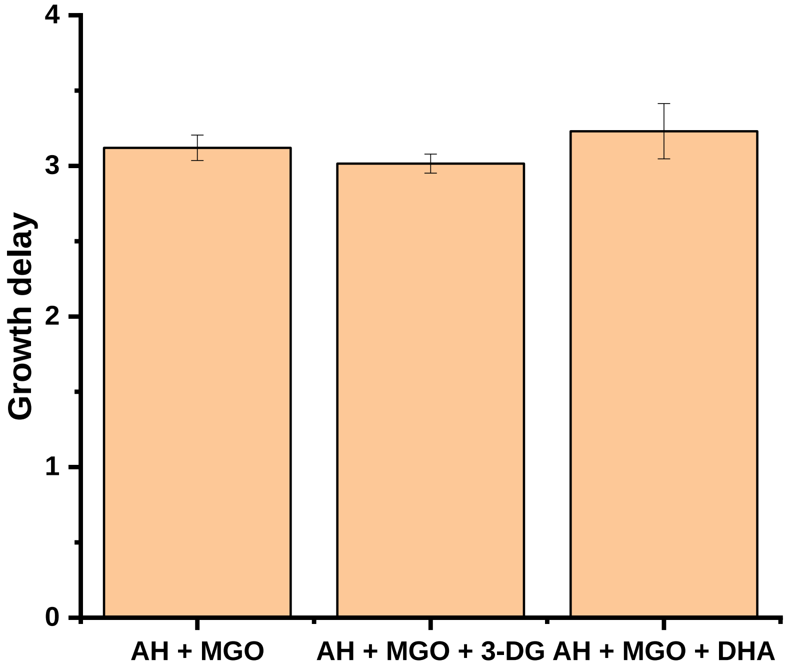

Figure S3. Growth delays of *B. subtilis* treated with 15 % (w/v, in LB medium) solutions artificial honeys, artificial honeys were spiked with 259 mg/kg MGO (AH+MGO) or 259 mg/kg MGO and 1620 mg/kg 3-deoxyglucosone (AH+MGO+3-DG) or 259 mg/kg MGO and 803 mg/kg dihydroxyacetone (AH+MGO+DHA). Measurements were carried out in duplicates. Error bars show standard deviation

Table S2. Raw data of figures 3 and 4: Growth delays of *B. subtilis* treated with 15 % solutions (w/v, in LB-medium) of artificial honey and Manuka honey 30+, respectively, honeys were spiked with MGO and 3-PLA. Measurements were carried out in quadruplicates. Measurement uncertainty shows standard deviation.

	**PLA spike**				
**MGO level**	**0 mg/kg**	**500 mg/kg**	**1000 mg/kg**	**1500 mg/kg**	**2000 mg/kg**
	Artificial honey				
0 mg/kg	1.00 ± 0.08	1.07 ± 0.06	1.06 ± 0.08	1.06 ± 0.04	1.03 ± 0.02
100 mg/kg	1.58 ± 0.02	1.61 ± 0.02	1.58 ± 0.07	1.62 ± 0.06	1.50 ± 0.13
250 mg/kg	1.90 ± 0.06	2.48 ± 0.06	2.52 ± 0.06	2.68 ± 0.09	2.86 ± 0.09
400 mg/kg	4.06 ± 0.11	4.45 ± 0.14	4.62 ± 0.35	4.83 ± 0.23	5.05 ± 0.32
	Manuka honey MH30+				
72 mg/kg	1.21 ± 0.06	1.28 ± 0.06	1.21 ± 0.06	1.27 ± 0.03	1.21 ± 0.05
172 mg/kg	1.95 ± 0.09	2.05 ± 0.11	2.21 ± 0.07	2.15 ± 0.05	1.95 ± 0.10
322 mg/kg	2.37 ± 0.11	3.17 ± 0.18	3.35 ± 0.12	3.46 ± 0.11	3.67 ± 0.12
472 mg/kg	5.64 ± 0.28	6.22 ± 0.54	6.41 ± 0.06	6.63 ± 0.22	6.85 ± 0.19

Table S3. Raw data of figure 5: MGO concentrations during the incubation (24 h at 37 °C) of 120 mg/L MGO solution in LB medium and in LB medium containing 600 mg/L 3-PLA. Measurements were carried out in triplicates. Measurement uncertainty shows standard deviation.

**Incubation time**	**MGO concentration [mg/L]**	
	**LB**	**LB + 600 mg/L 3-PLA**
0 h	121.7 ± 2.2	129.3 ± 0.6
1 h	103.3 ± 2.5	117.2 ± 4.4
3 h	61.7 ± 1.2	99.9 ± 5.6
5 h	53.1 ± 2.6	92.6 ± 1.0
8 h	39.3 ± 0.8	67.4 ± 0.8
24 h	7.3 ± 0.6	35.3 ± 0.2

Table S4. Raw data of figure 4: Growth delays of *B. subtilis* treated with 15 % solutions (w/v, in LB-medium) of artificial honey, honeys were spiked with MGO and gallic acid. Measurements were carried out in duplicates. Measurement uncertainty shows standard deviation.

	**Gallic acid spike**					
**MGO level**	**0 mg/kg**	**100 mg/kg**	**500 mg/kg**	**1000 mg/kg**	**1500 mg/kg**	**2000 mg/kg**
0 mg/kg	1.00 ± 0.00	0.98 ± 0.02	0.98 ± 0.02	0.98 ± 0.02	0.98 ± 0.02	0.95 ± 0.02
100 mg/kg	1.60 ± 0.01	1.57 ± 0.10	1.64 ± 0.06	1.60 ± 0.06	1.64 ± 0.10	1.70 ± 0.02
250 mg/kg	3.41 ± 0.10	3.54 ± 0.19	3.62 ± 0.12	3.79 ± 0.11	4.06 ± 0.13	4.17 ± 0.19
400 mg/kg	4.94 ± 0.13	4.92 ± 0.25	5.41 ± 0.19	5.48 ± 0.24	5.66 ± 0.10	no growth

Table S5. Growth delays of *B. subtilis* treated with artificial honey spiked with 259 mg/kg MGO (AH+MGO), artificial honey spiked with 259 mg/kg MGO and 467 mg/kg PLA (AH+MGO+PLA) and commercial MH250+ manuka honey with 259 mg/kg MGO and 467 mg/kg PLA (MH250+) in dependence on the MGO content in the assay. Measurements were carried out in triplicates. Measurement uncertainty shows standard deviation.

MGO	AH+MGO	AH+MGO+3-PLA	MH250+
6.83 µg/mL	1.13 ±0.03	1.14 ± 0.03	1.28 ± 0.04
13.67 µg/mL	1.51 ± 0.12	1.44 ± 0.06	2.10 ± 0.32
20.50 µg/mL	2.22 ± 0.01	2.14 ± 0.08	3.88 ± 0.40
27.33 µg/mL	3.20 ± 0.12	3.41 ± 0.11	6.20 ± 0.32
34.16 µg/mL	3.92 ± 0.22	4.27 ± 0.12	7.71 ± 0.33
41.00 µg/mL	4.94 ± 0.15	5.45 ± 0.15	no growth
